# Why Is the Negro Black?

**Published:** 1896-11

**Authors:** 


### WHY IS THE NEGRO BLACK?

This question is propounded by *The Hospital*, which then proceeds to answer it as follows, basing its statements on an article in an unnamed American contemporary:

"It has occurred to a writer in an American medical newspaper to discuss the blackness of the negro's skin. It will be a revelation to many to learn that the baby negro is not born black. Even so long ago as 1765 LeCat noticed that the newly born negro is of a reddish color. That observation has since been frequently confirmed; and it is now pretty widely known that the baby negro begins to follow in the footsteps of his parents as regards color within a few days after birth, yet at the moment of birth he shows a disposition to aspire toward the civilized races, being white, or, at worst, red in hue. It is generally assumed that the primest of all the causes of a negro's blackness is the hot sun beneath whose more or less vertical rays he is doomed to live. There is, however, a physiological condition of the skin which differentiates that organ from the integument of an European. 'The negro,' says our American scientist, 'possesses a more developed vascular sudoriparous system than we do.' In other words, he has more and larger sweat glands, and they are more liberally supplied with blood. By means of these he perspires much more abundantly. This condition is possibly a contributory factor in his blackness. It is an important element in the investigation to remember, however, that the blackest of all black people are almost invariably found under certain very definite climatic conditions. That is to say, they are found where great heat, strong light, and much atmospheric moisture are in combination. For example, 'the blackest negroes in Africa are those who live in Guinea, where the greatest amount of rain annually falls.' On the other hand, 'the people who live in the dry section of the Nubian Desert have red skins. Heat, light, and humidity are all causes of pigmentation, and if to these we add the fact of the highly 'developed vascular sudoriparous system of the negro,' we have traveled as far as our American investigator is able to help us. The question is one of genuine scientific interest; and, perhaps, when the Matabele, and the Dervishes, and the Soudanese have all settled down quietly in the ways of civilization and order, science may turn her attention in this direction, and tell us much that is both new and interesting about those races who differ so markedly from ourselves in color, character and many other particulars."

